# Deregulation of Stemness-Related Genes in Endometriotic Mesenchymal Stem Cells: Further Evidence for Self-Renewal/Differentiation Imbalance

**DOI:** 10.29252/ibj.24.5.328

**Published:** 2020-04-18

**Authors:** Parisa Mashayekhi, Mehrdad Noruzinia, Sepideh Khodaverdi

**Affiliations:** 1 ‍‍Department of Medical Genetics, Faculty of Medical Sciences, Tarbiat Modares University, Tehran, Iran;; 2 Endometriosis Research Center, Iran University of Medical Science, Tehran, Iran

**Keywords:** Endometriosis, Mesenchymal stem cells, Transcription factors

## Abstract

**Background:**

Any irregularities in self-renewal/differentiation balance in endometriotic MSCs can change their fate and function, resulting in endometriosis development. This study aimed to evaluate the expression of *OCT4 *transcripts (*OCT4A*, *OCT4B*, and *OCT4B1*), *SOX2*, and *NANOG* in endometriotic MSCs to show their aberrant expression and to support self-renewal/differentiation imbalance in these cells.

**Methods:**

MSCs were isolated from three endometriotic and three normal endometrium samples and characterized and analyzed for the expressions of *OCT4A*, *OCT4B*, *OCT4B1*, *SOX2*, and *NANOG* using the qRT-PCR.

**Results:**

The expressions of *OCT4 transcripts *and *NANOG* increased significantly in endometriotic MSCs, whereas *SOX2* expression did not show any significant difference.

**Conclusion:**

Our findings provide further evidence for confirming the self-renewal/ differentiation imbalance in endometriotic MSCs, as the main underlying cause of endometriosis development. This study also paves the way for further research on endometriosis treatment by focusing on endometriotic stem cells.

## INTRODUCTION

Endometriosis is a common debilitating gynecologic disorder recognized by the presence of endometrial tissue (gland and stroma) outside the uterus, especially in pelvic organs^[^^1 ▶^^]^. Clinically, the disease is mostly manifested with pelvic pain, painful intercourse, and infertility^[^^2 ▶^^]^. Retrograde menstruation is the oldest principle in endometriosis pathogenesis, and stem cell theory is the most popular issue. During each menses, stem cells are transmitted to peritoneum through retrograde menstruation^[^^3 ▶^^]^. Endometriosis occurs in 10% of females^[^^4 ▶^^]^, while retrograde menstruation appears in most women^[^^5 ▶^^]^; this fact highlights the different characteristics of endometriotic MSCs. Self-renewal and differentiation are essential factors in determining stem cell fate^[^^6 ▶^^]^, and any disturbance in self-renewal/differentiation equilibrium can alter the characteristics and functions of stem cells, thereby causing various kinds of diseases. Self-renewal/ differentiation imbalance is likely to be present in endometriotic MSCs, which could be considered as the underlying reason for endometriosis. In endometriotic women, eutopic endometrial cells have shown increased proliferation activity^[^^7 ▶^^]^ and decreased differentiation/decidualization capacity^[^^8 ▶^^]^. Several factors, including transcription factors, epigenetic regulatory factors, and miRNAs, influence the self-renewal/differentiation balance in stem cells^[^^9 ▶^^]^. 

miRNAs are epigenetic factors deregulated in endometriotic MSCs^[^^10 ▶^^]^ and can serve as biomarkers in the early diagnosis of endometriosis^[^^11 ▶^^]^. Transcription factors *OCT4*,* SOX2*, and *NANOG* organize the core pluripotency network, which in turn regulates the pluripotency of stem cells. These factors are naturally expressed in both embryonic and adult stem cells^[^^12 ▶^^,^^13 ▶^^]^. They also participate in pathways that organize a mutual regulatory circuit with epigenetic regulatory factors like miRNAs to balance self-renewal/proliferation and differentiation of stem cells^[^^14 ▶^^]^. Thus, their deregulation can disturb the stem cells balance and change their fate.

The human *OCT4* gene can produce three transcripts (*OCT4A*, *OCT4B*, and *OCT4B1*) by alternative plicing^[^^15 ▶^^,^^16 ▶^^]^. *OCT4A *is the most studied and described isoform, and its fine-tuning is necessary for maintaining pluripotency or inducing differentiation in stem cells^[^^17 ▶^^]^. During the differentiation process, the expression levels of *OCT4A* are decreased^[^^18 ▶^^]^. In tumor cell lines, *OCT4B* modulates *OCT4A* expression as a non-coding RNA, and its overexpression enhances the expression of *OCT4A *and cell proliferation and decreases apoptosis^[^^19 ▶^^]^. Ectopic expression of *OCT4B* promotes cell proliferation, migration, and angiogenesis in addition to the suppression of caspase activity^[^^20 ▶^^,^^21 ▶^^]^ and its mediated differentiation^[^^22 ▶^^]^. *OCT4B1* is mainly expressed in pluripotent cells^[^^23 ▶^^]^ and is downregulated following differentiation^[^^15 ▶^^]^. There is a direct relationship between the expression of *OCT4B1* and stemness-related genes (*OCT4*,* SOX2*,* NANOG*, and *KLF4*), and its downregulation in cancer cell lines inhibits the expression of these genes^[^^24 ▶^^]^. *OCT4B1 *overexpression also accelerates cell proliferation^[^^24 ▶^^]^ and has anti-apoptotic effects via suppressing caspase activity^[^^25 ▶^^]^. Caspase is a class of proteases involved in apoptosis, and its activity is essential for cell differentiation^[^^22 ▶^^]^. Fine-tuning intensity or duration of caspase signaling is vital in the death/differentiation cell fate decision^[^^26 ▶^^]^, therefore, *OCT4B* and *OCT4B1* can influence the differentiation of stem cells by fine-tuning of the caspase activity. 

It seems that the aberrant expression of stemness-related genes disturbs the self-renewal/differentiation balance of endometriotic MSCs in favor of increased proliferation and migration and reduced differentiation, which contributes to endometriosis development. Considering this hypothesis, we evaluated the expression levels of *OCT4A*, *OCT4B*, *OCT4B1*, *SOX2*, and *NANOG* in endometriotic MSCs and compared them with normal endometrial MSCs. 

## MATERIALS AND METHODS

**Specimen collection**

Human endometrial tissue samples were obtained from women aged 30–45 years (mean 34.8 ± 4.7) in the secretory phase. Three women were healthy and volunteered for the study, and another three women underwent laparoscopic surgery for stages III and IV endometriosis in the Rasoul Akram Hospital, Tehran, Iran. None of the women had received hormone therapy for at least three months before surgery. 

**Isolation and culture of human endometrial MSCs**

Human endometrial tissue was isolated from the myometrium. Collected tissue was dissociated into single-cell suspensions by mechanical methods and using enzymatic digestion by collagenase type 3 (300 µg/ml; Sigma, Germany) at 37°C for 90 minutes. Endometrial stromal cells were cultured in T25 culture flasks containing DMEM/Ham's F-12 (Invitrogen, UK), 1% penicillin-streptomycin solution, and 10% FBS (Gibco, USA). 

**Flow cytometry analysis of endometrial stromal cells**

Endometrial stromal cells in passages 3-4 were harvested and characterized using flow cytometry for cell surface markers. Cells were stained with phycoerythrin- or fluorescein isothiocyanate-conjugated antibodies. Anti-human CD90, CD73, and CD146 (all from BD Bioscience, USA), as well as CD105 (Immunostep, Spain) were used as specific antibodies for MSCs and anti-human CD45 (BD Bioscience) and CD34 (Immunostep) as specific antibodies for hematopoietic stem cells, served for negative controls. Cells were analyzed using a FACS calibur apparatus (Becton Dickinson, USA), and the collected data were analyzed using FlowJo 7.6 software.

**Differentiation of endometrial MSCs**

To characterize, the endometrial stromal cells were seeded in 24-well plates and cultured for three weeks in osteogenic and adipogenic differentiation media, separately. Cells cultured in low-serum medium (DMEM/F12 with 1% each of FBS and antibiotic) were used as the control group. The medium was changed every 2-3 days. After three weeks, the stromal cells were fixed and stained with 4% Alizarin Red (pH 4.1) and 1% Oil Red O (both from Sigma) to assess osteogenic and adipogenic differentiation.

**Gene expression analyses**

Total cellular RNA was collected from cultured cells using TRIzol Reagent (Sigma). Reverse transcription of the extracted RNA was performed with the cDNA synthesis kit (Takara Bio, USA, Inc.). Specific primers for the *OCT4 *splice variants (*OCT4A*, *OCT4B*, and *OCT4B1*), as shown in Table 1, were designed as described before by Atlasi *et al.*^[15]^. Also, in order to evaluate *SOX2*,* NANOG*, and *GAPDH *(as an internal control) expression levels, the specific primers sets were designed using Allele ID6 and Oligo7 software (Table 1). The qRT-PCR was carried out in an AB StepOne thermocycler using the SYBR Green qPCR Master Mix (Applied Biosystems, USA), according to the manufacturer's protocol. PCR efficiency was determined using LinReg software. The expression level of each target gene was normalized in reference to the *GAPDH* mRNA level and analyzed using the Pfaffl method. 

**Table 1:** Sequences of oligonucleotides used for real-time PCR

**Primer name**	**Sequence (5' → 3')**
*GAPDH*-F	ATGAGAAGTATGACAACAGCCTC
*GAPDH*-R	CATGAGTCCTTCCACGATACC
*OCT4A*-F	CTTCTCGCCCCCTCCAGGT
*OCT4A*-R	AAATAGAACCCCCAGGGTGAGC
*OCT4B*-F	AGACTATTCCTTGGGGCCACAC
OCT4B-R	GGCTGAATACCTTCCCAAATAGA
*OCT4B1*-F	AGACTATTCCTTGGGGCCACAC
*OCT4B1*-R	CTTAGAGGGGAGATGCGGTCA
*SOX2*-F	AGTATCAGGAGTTGTCAAGGC
*SOX2*-R	CTGGGGCTCAAACTTCTCTC
*NANOG*-F	CCTCTATACTAACATGAGTGTGG
*NANOG*-R	CATGGAGGAAGGAAGAGGAGA

**Statistical analysis**

The data were analyzed by *t*-test using GraphPad Prism 6 software, and results with *p* values of less than 0.05 were considered statistically significant.

**Ethical statement**

The above-mentioned sampling protocols were approved by the Ethics Committee of Medical Faculty of Tarbiat Modares University, Tehran, Iran (ethical code: 1395.409). Written informed consents were obtained from all the women who are participated in this study.

## RESULTS

**Characterization of endometrial MSCs**

**Fig. 1:**
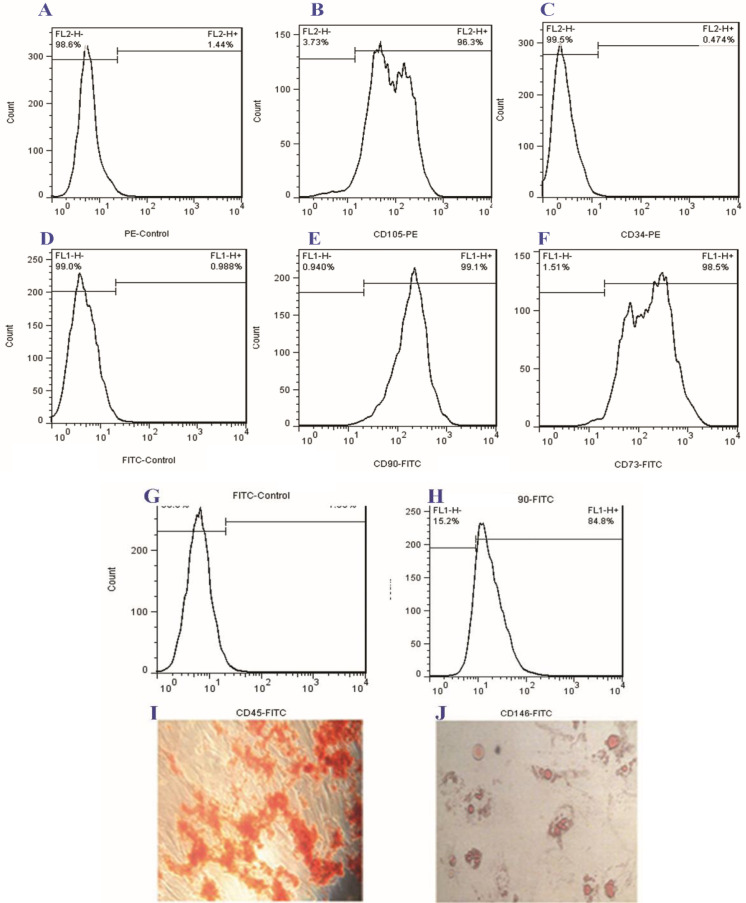
Isolation and characterization of endometrial MSCs. (A) phycoerythrin-conjugated isotype control, (B) CD105 (96.3%), (C) CD34 (0.474%), (D) fluorescein isothiocyanate-conjugated isotype control, (E) CD90 (99.1%), (F) CD73 (98.5%), (G) CD45 (1.99%), and (H) CD146 (84.8%), and (I) osteogenic and (J) adipogenic differentiation of isolated endometrial MSCs

The expressions of mesenchymal markers CD73 (98.5%), CD90 (99.1%), CD105 (96.3%), and CD146 (84.8%) were confirmed by flow cytometry analysis. The expressions of hematopoietic markers CD34 (0.474%) and CD45 (1.99%) were negative in isolated cells (Fig. 1A-1H). Adipogenic and osteogenic differentiation was induced in cultured cells with specific differentiation media, then the result was visualized by Alizarin Red staining and Oil Red staining for calcium deposits and lipid vacuoles, respectively (Fig. 1I and 1J).

**Upregulation of** ***OCT4*** **transcripts in endometriotic MSCs**

**Fig. 2:**
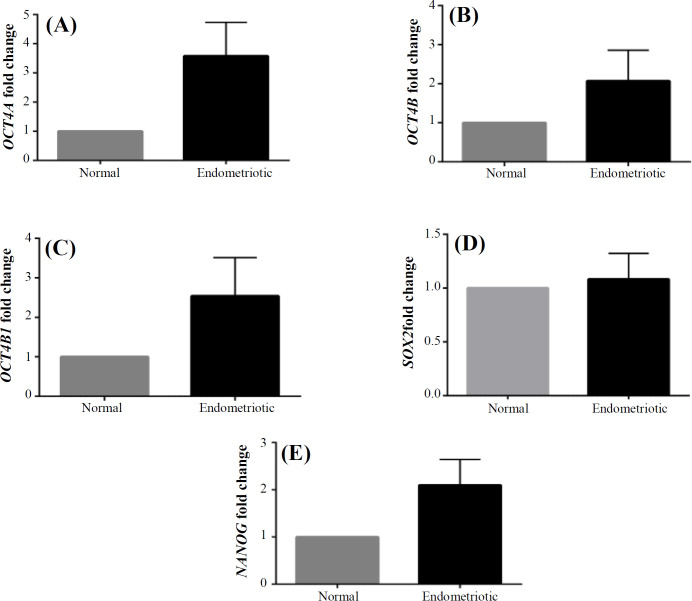
Relative expressions of* OCT4A* (A),* OCT4B* (B),* OCT4B1* (C), *SOX2* (D), and *NANOG* (E) in endometriotic MSCs in comparison with normal ones, detected by RT-PCR

Relative expressions of *OCT4A* showed the upregulation of this transcript (3.58 ± 1.11, *p* < 0.001) in endometriotic MSCs (Fig. 2A). *OCT4B* and *OCT4B1* expression levels were 2.00 ± 0.78 (*p* = 0.007) and 2.54 ± 0.97 (*p* = 0.003) fold higher than the normal MSCs, respectively (Fig. 2B and 2C).

***SOX2*** **and** ***NANOG*** **expressions in endometriotic MSCs**

*SOX2* expression in endometriotic MSCs did not show any significant difference (1.08 ± 0.05; *p *= 0.1) relative to the normal MSCs (Fig. 2D), but a significant increase was observed in the expression of NANOG (2.00 ± 0.57; *p* = 0.001; Fig. 2E).

## DISCUSSION

Stem cell theory is the most favored theory in endometriosis pathogenesis. It seems that endo-metriotic MSCs are different from normal ones and have impaired self-renewal/differentiation balance. The aberrant expression of transcription factors (*OCT4*, *SOX2*, and *NANOG*) can disturb the balance in stem cells and alter their function. Ectopic expression of these transcription factors in cancer stem cells is correlated with poor differentiation, large tumor size, and high-grade tumor^[^^27 ▶^^]^. Co-expression of *OCT4* and *NANOG* in lung adenocarcinoma enhances cell proliferation and motility and decreases their differentiation by inducing cancer stem cell-like properties^[^^28 ▶^^]^. 

In this study, we investigated for the first time the expression of *OCT4* transcripts (*OCT4A*, *OCT4B*, and *OCT4B1*), together with* SOX2* and *NANOG *in endometriotic MSCs and found that *OCT4A* upregulated as compared to the healthy controls. Previous studies have demonstrated the upregulation of *OCT4A* in the eutopic and ectopic endometrium of women with endometriosis^[^^29 ▶^^,^^30 ▶^^]^. Overexpression of *OCT4A *mRNA enhanced self-renewal, tumorsphere generation capacity, cell motility, and invasion of medulloblastoma cells^[^^31 ▶^^]^. However, *OCT4A *decreases in stem cells during their differentiation^[^^18 ▶^^]^. The increased expression of *OCT4A *in our study might increase self-renewal and migration in stem cells and decreas the differentiation potential. 

Our findings demonstrated that *OCT4B* upregulated in endometriotic MSCs. *OCT4B* regulated *OCT4A* expression by competing for endogenous RNA in a miRNA-dependent manner in tumor cells, resulting in the increased cell proliferation and self-renewal and decreased cell apoptosis^[^^19 ▶^^]^. *OCT4B* elevates epithelial-mesenchymal transition and cell migration^[^^32 ▶^^]^. Therefore, it is likely that the increased *OCT4B* expression in our study can promote self-renewal migration of stem cells and suppress caspase activity, which leads to the decreased differentiation and improved survival of migrated stem cells. 

Our results suggested the increased expression of *OCT4B1* in endometriotic MSCs. *OCT4B1* is in a close relationship with pluripotency regulator genes, and its downregulation causes the decreased expression of *OCT4A*,* SOX2*,* NANOG*, and *KLF4*^[^^24 ▶^^]^. *OCT4B1 *enhances the proliferation and the growth of colon cancer cells by maintaining stem cell properties. Furthermore, it increases cell migration and reduces apoptosis^[^^33 ▶^^]^. *OCT4B1* downregulation significantly raises the activity of caspase-3 and -7 and elevates the apoptosis rate in gastric cancer cells^[^^25 ▶^^]^. It seems that the increased expression of *OCT4B1 *in this study is in favor of elevating self-renewal and the migration of endometrial MSCs in addition to decreasing their differentiation through suppressing caspase activity.

*SOX2* is one of the critical transcription factors involved in stem cell fate. In this study, we did not observed any significant increase in the mRNA level of *SOX2* in endometriotic MSCs. 

Herein, *NANOG* expression raised in endometriotic MSCs. A former study also found an increase in *NANOG* expression in the eutopic and ectopic endometrium of endometriotic women^[^^30 ▶^^]^. Down-regulation of *NANOG* in human hepatocellular carcinoma decreases the expression of *SOX2*,* OCT4*, and *KLF4*, giving rise to the reduced proliferation, invasion, and migration of cancer cells^[^^34 ▶^^]^. *NANOG* and *OCT4* suppress the expression of genes that were vital for differentiation, by recruiting repressive complexes of the Polycomb-group proteins^[^^35 ▶^^,^^36 ▶^^]^. In our study, the increased expression of *NANOG* in endometriotic MSCs probably disturbs the stem cell balance by increasing the proliferation and migration and also by repressing their differentiation.

Although there is no consensus on the cause of endometriosis, evidence highlights the role of stem cells in endometriosis development^[^^3 ▶^^]^ and confirms their different characteristics and functions^[^^8 ▶^^,^^37 ▶^^]^. Our findings augmented the theory of self-renewal/ differentiation imbalance in endometriotic MSCs, which results in endometriosis development and shows their varied characteristics. Our study also helps clraify the etiology of this complex disease and pave the way for further research on endometriosis treatment by focusing on stem cells.
